# The Employment of Blisters in Acute Diseases of the Brain

**Published:** 1846-02

**Authors:** 


*The employment of Blisters in acute diseases of the Brain.* By Dr. Tritschler.—Blisters are frequently used and applied to all parts of the body except the frontal region, which, according to Dr. T., is the best place to make them act in acute affections of the brain. He covers the whole frontal region, and even the root of the nose, by a vesicatory, and remarks, that besides a free suppuration produced by it, there is generally a copious flow of mucous from this organ. We learn, too, from the Journal des Connaisances Medico-Chirurgicales, that the physicians in the Parisian Hospitals employ these blisters with success against cerebral symptoms in severe fevers. The application of a single one is not sufficient to disfigure a patient.—*Southern Med. and Surg. Jour.*

